# The Effects of Different Respiratory Viruses on the Oxidative Stress Marker Levels in an In Vitro Model: A Pilot Study

**DOI:** 10.3390/ijms252212088

**Published:** 2024-11-11

**Authors:** Barbara Bażanów, Katarzyna Michalczyk, Alina Kafel, Elżbieta Chełmecka, Bronisława Skrzep-Poloczek, Aleksandra Chwirot, Kamil Nikiel, Aleksander Olejnik, Alicja Suchocka, Michał Kukla, Bartosz Bogielski, Jerzy Jochem, Dominika Stygar

**Affiliations:** 1 Faculty of Veterinary Medicine, Department of Pathology, Division of Microbiology, Wrocław University of Environmental and Life Sciences, 50-375 Wrocław, Poland; 2 Department of Physiology in Zabrze, Faculty of Medical Sciences in Zabrze, Medical University of Silesia in Katowice, 40-055 Katowice, Poland; 3 Department of Natural Sciences, Institute of Biology, Biotechnology and Environmental Protection, University of Silesia in Katowice, 40-007 Katowice, Poland; 4 Department of Medical Statistics, Faculty of Pharmaceutical Sciences in Sosnowiec, Medical University of Silesia, 40-055 Katowice, Poland; 5 Department of Internal Medicine and Geriatrics, Faculty of Medicine, Jagiellonian University Medical College, 31-688 Kraków, Poland; 6 Department of Endoscopy, University Hospital, 30-688 Kraków, Poland

**Keywords:** human adenovirus, human coronavirus, human rhinovirus, in vitro model, lung carcinoma A549 cells, lung fibroblast MRC-5 cells, oxidative stress markers, respiratory virus

## Abstract

Respiratory viruses are among the most common causes of human infections. Examining pathological processes linked to respiratory viral infections is essential for diagnosis, treatment strategies, and developing novel therapeutics. Alterations in oxidative stress levels and homeostasis are significant processes associated with respiratory viral infections. The study aimed to compare selected oxidative stress markers: total oxidative status (TOS), total antioxidant capacity (TAC), and the oxidative stress index (OSI) levels and glutathione peroxidase (GPx) and glutathione reductase (GR) activities in normal (MRC5 cell line) and tumor (A549 cell line) lung cells infected with human coronaviruses (HCoV) OC43 and 229E, human adenovirus type 5 (HAdV5), or human rhinovirus A (HRV A). We observed that a respiratory viral infection more significantly affected non-enzymatic oxidative stress markers in a lung adenocarcinoma model (A549 cells), while human lung fibroblasts (MRC-5 cell line) presented changes in enzymatic and non-enzymatic oxidative stress markers. We suggest that further detailed research is required to analyze this phenomenon.

## 1. Introduction

Respiratory viruses are among the most common causes of human infections. Despite the development of prevention and therapy, the high morbidity and mortality caused by these viruses constitute a significant problem and challenge for modern medicine. Infections in immunocompromised, chronically ill, elderly, or pediatric patients generally have a more severe course and are characterized by a higher number of deaths. It is estimated that about one-fifth of child deaths worldwide are caused by acute respiratory infections [1].

The presented research focuses on four infectious agents, the typical respiratory viruses that cause the common cold: human coronaviruses (CoV) OC43 and 229E, adenovirus type 5 (HAdV 5), and human rhinovirus A (HRV A). Coronavirus infections (*Coronaviridae*, RNA viruses) are usually mild or subclinical and generally self-limiting. However, in high-risk groups, they are associated with a range of respiratory complications, including bronchiolitis and pneumonia [2]. These viruses have a distinct winter seasonality between December and April, and they are not diagnosed during the summer months [3]. Human adenovirus 5 (*Adenoviridae*, DNA virus) is one of the adenovirus serotypes that can infect humans. It is responsible for a wide range of respiratory, gastrointestinal, and ocular infections in humans [4]. It can cause more severe disease, especially in people with weakened immune systems.

The body’s immune response to infection with the human rhinovirus (*Picornaviridae*, RNA virus) is usually sufficient to eliminate the virus. However, the acquired immunity does not protect against subsequent infections with rhinovirus due to many different strains of HRV. For this reason, an effective rhinovirus vaccine has not yet been developed [5].

The accumulation of reactive oxygen species (ROS) affects the host’s defense mechanisms, which in turn enhances viral infections. Reactive oxygen species (ROS) are also produced in the infected cells due to viral infections. It has been demonstrated that antioxidant systems shield host cells against a range of viruses by altering their oxidative/antioxidative status [6]. Reactive oxygen species have the ability to eliminate infections either directly through oxidative damage to biocompounds or indirectly through various non-oxidative methods [7]. However, it is unclear which alterations in metabolic parameters and pathways contribute to producing reactive oxygen species (ROS) during viral infections.

Numerous disorders, including cancer, degenerative diseases, metabolic diseases, and aging, are related to oxidative stress [8]. Reactive oxygen species have the potential to be pro- or anti-carcinogenic depending on how they enhance angiogenesis, proliferation, invasiveness, angiogenesis, and metastasis while also suppressing apoptosis. Examples of ROS anti-carcinogenic effects include increasing cell-cycle stasis, apoptosis, and necrosis [8]. Research has also demonstrated the involvement of oxidative pathways in the start, maintenance, and advancement of carcinogenesis [9].

Many papers published during and after the COVID-19 (coronavirus disease 2019) pandemic analyzed the severity of SARS-CoV-2 (severe acute respiratory syndrome coronavirus 2) infection in previously healthy patients and patients with primary lung diseases, like lung cancer patients. Luo et al. [10] found that infection in cancer patients was associated with its severe clinical course, with 62% of patients requiring hospitalization and 25% of cases ending in death. Also, according to Hajjar et al. [11], infection with the H1N1 influenza virus in patients with various types of lung diseases, such as cancer, can induce a severe illness, leading to acute respiratory distress syndrome and death [11]. Respiratory viruses belong to different families and have different structures and genomes. They differ in the course of infection, the susceptibility of populations, the seasonality of circulation, and the mode and ease of transmission.

Cells express several physiological defenses against intracellular oxidative stress, including cellular antioxidant enzymes glutathione reductase (GR) and glutathione peroxidase (GPx) [12]. However, values of individual oxidative stress markers do not correctly reflect the oxidative stress level. The correct assessment includes the calculation of the oxidative stress index (OSI), which measures both total oxidant status (TOS) and total antioxidant capacity (TAC) [13].

Examining pathological processes linked to respiratory viral infections is essential for diagnosis, treatment strategies, and developing novel therapeutics. Alterations in oxidative stress marker homeostasis are significant processes associated with respiratory viral infections, contributing to inflammation and consequent tissue destruction. The presented pilot study aimed to investigate the levels of oxidative stress markers in lung cells caused by different respiratory viruses causing the common cold. The aim of the study was to determine the differences in oxidative stress parameters induced by viral infection in healthy vs. cancer cells. Due to the more severe course of infection in at-risk individuals, we aimed to compare selected oxidative stress markers on both normal (MRC5 cell line, isolated from the normal lung tissue of a male embryo) and tumor (A549 cell line, isolated from the lung tissue of a Caucasian, male, lung cancer patient) lung cells infected with human coronaviruses (HCoV) OC43 and 229E, human adenovirus type 5 (HAdV5), or human rhinovirus A (HRV A). Comparative studies may serve as a foundation for examining the response of lung tissue to respiratory virus infections in both healthy individuals and those with lower respiratory tract diseases. This understanding is essential for elucidating the pathological mechanisms involved in these infections and for developing more effective therapeutic approaches.

## 2. Results

### 2.1. Oxidative Stress Markers in the Lung Carcinoma A549 Cell Line

**Figure 1:**
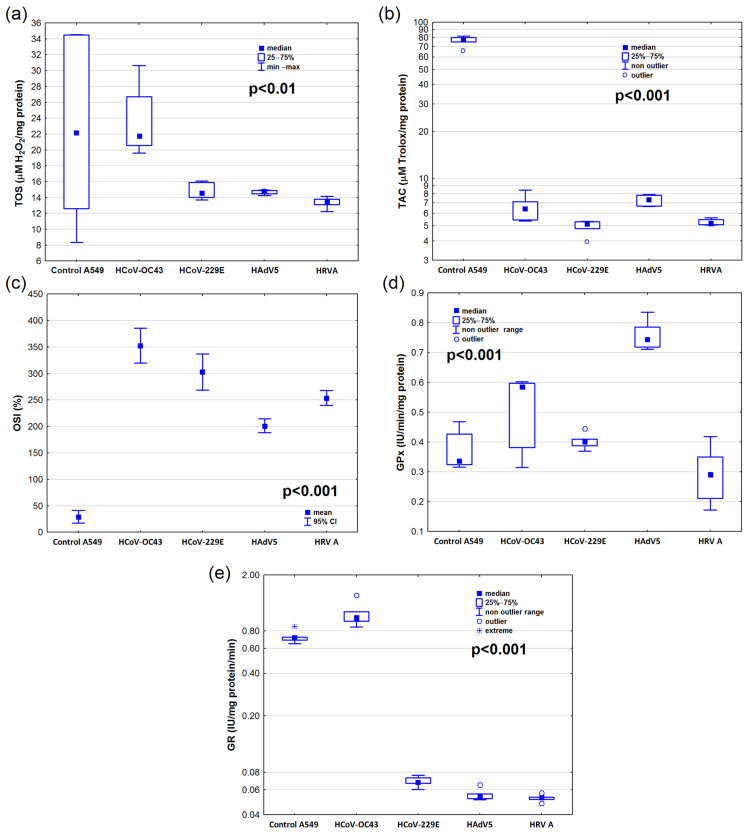
Oxidative stress marker levels: (**a**) the total oxidative status (TOS), (**b**) total antioxidant capacity (TAC), (**c**) oxidative stress index (OSI), (**d**) glutathione peroxidase (GPx) activity, and (**e**) glutathione reductase (GR) activity in the cells infected with different respiratory viruses and in control cells of the human lung carcinoma cell line (A549). The results are presented as Me (Q_1_; Q_3_)—median (lower-upper quartile) or M ± SD—mean ± standard deviation. For better visualization in part (**b**,**e**), the logarithmic scale was used. Abbreviations: HAdV5—human adenovirus 5; HcoV-229E—human coronavirus 229E; HcoV-OC43—human coronavirus OC43; HRV A—human rhinovirus A; IU—international activity unit.

We observed statistically significant differences in TOS, TAC, OSI values, and GPx and GR activities depending on the type of viral infection (Figure 1, Table S1). However, we noted no statistically significant differences in TOS values between the infected and A549 cells from the control group (Figure 1a). Total antioxidant capacity values were lower in all analyzed infected cells when compared to cells from the control group in the A549 cell line, but significant changes were found between the control and HCoV-229E (*p* < 0.001) and the control vs. HRV A (*p* < 0.001) (Figure 1b, Table S1).

We observed statistically significant differences in OSI values for infected cells (for all groups *p* < 0.001). The lowest OSI values were observed for the A549 cells from the control group, while the highest values were observed in the cells infected with HcoV-OC43 (Figure 1c, Table S1).

The highest GPx activity was observed in the cells infected with HAdV5 (Figure 1d), and it was significantly different from the A549 cells from the control group (*p* < 0.05). We noted no statistically significant differences in GPx activity between other infected cells and the A549 cells from the control group (Table S1).

The lowest GR activity in comparison to the control was observed in the cells infected with HCoV-229E, HAdV5 (Figure 1e).

### 2.2. Oxidative Stress Markers in Lung Fibroblast MRC-5 Cell Line

**Figure 2:**
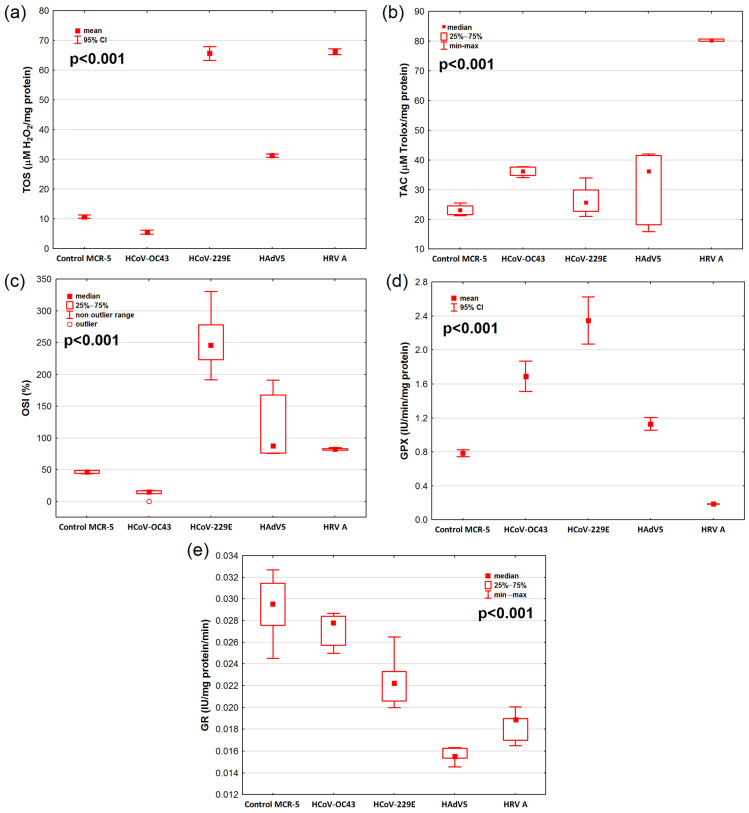
Oxidative stress marker levels: (**a**) the total oxidative status (TOS), (**b**) total antioxidant capacity (TAC), (**c**) oxidative stress index (OSI), (**d**) glutathione peroxidase (GPx) activity, and (**e**) glutathione reductase (GR) activity in the cells infected with different respiratory viruses and in control cells of the human lung carcinoma cell line (MCR-5). The results are presented as Me (Q_1_; Q_3_)—median (lower-upper quartile) or M ± SD—mean ± standard deviation. Abbreviations: HAdV5—human adenovirus 5; HcoV-229E—human coronavirus 229E; HcoV-OC43—human coronavirus OC43; HRV A—human rhinovirus A; IU—international activity unit.

The analysis of the results showed that the viral infection significantly affected TOS, TAC, and OSI values and GPx and GR activities (Figure 2, Table S2).

Viral infection significantly increased TOS values when compared to the MCR-5 cells from the control group (Figure 2a). The highest TOS values were observed for the cells exposed to HCoV-229E and HRV A virus, while the lowest TOS values were observed in the cells exposed to HCoV-OC43. The levels of TOS differed significantly between all virus groups and the control group (*p* < 0.001 in all cases, Table S2).

The highest TAC values were observed in cells exposed to HRV A, and they were 3.6 times higher compared to the MRC-5 cells from the control group. Similarly, TAC values in the HCoV-229E-infected cells were significantly higher compared to the MRC-5 cells from the control group (Table S2).

Statistically significant differences were demonstrated in OSI values depending on the virus used (*p* < 0.001) in the cell line MCR-5 (Figure 2c).

The results showed that GPx activity was significantly higher in cells exposed to HCoV-OC43, HCoV-229E, and HAdV5 and significantly lower in cells exposed to HRV A (*p* < 0.001) when compared to the MRC-5 cells from the control group (Figure 2d, Table S2).

The lowest GR activity was observed in cells exposed to HAdV5, and it was significantly lower (*p* < 0.001) than GR activity in MRC-5 cells from the control group. GR activity in the rest of the infected cells was similar and did not differ from the MRC-5 cells from the control group (Figure 2e, Table S2).

## 3. Discussion

In the presented study, we have analyzed the effects of respiratory virus infection on the oxidative stress markers in an in vitro model of human lung carcinoma (A549) and human lung fibroblast (MCR5) cell lines. The presented results show that HCoV infection caused greater changes in non-enzymatic markers and HAdV5 and HRV A infections in enzymatic antioxidative stress markers. An imbalance in the oxygen reactive species (ROS) production and the body’s inability to detoxify them is referred to as oxidative stress [14]. Oxidative stress might be triggered by a wide variety of viral infections: HIV 1; hepatitis viruses B, C, and D; herpes viruses; and respiratory viruses, such as coronaviruses [15]. In addition, its intensity is indicative of the infection severity and outcome. Although it has been clearly understood that reactive oxygen and nitrogen species production causes lung tissue injury and epithelial barrier dysfunction in the course of viral, bacterial, and parasitic infections, the molecular inflammation mechanisms of oxidative stress contributing to infection progression still need to be researched to improve therapy management in patients [16]. Human coronavirus 229E (HCoV-229E), SARS-CoV-2, SARS-CoV, Middle East respiratory syndrome coronavirus (MERS-CoV), HCoV-OC43, HCoV-NL63, and HKU-1 are the seven human coronaviruses (HCoVs) that have been found to date. Acute respiratory distress syndrome (ARDS) or multi-organ dysfunction is a known side effect of SARS-CoV-2, SARS, and MERS, particularly in older adults with comorbidities [17]. Human coronaviruses OC43, NL63, 229E, and HKU-1 are four of them that usually only cause minor respiratory infections in people.

Mitochondrial dysfunction can underlay hypoxia induced by lung injury. Eventually, it may lead to an increase in ROS production and a relative decrease in oxygen and energy production. H_2_O_2_ and other reactive species produced by the mitochondrial respiratory chain enhance the expression of many genes that prompt macrophages, neutrophils, and endothelial cells to produce proinflammatory cytokines, which enhance the production of other superoxides and H_2_O_2_ [18].

Elevated ROS levels in the early stages of viral infections adversely affect the cells and tissues and are important for antiviral immune function [19]. Studies show that the deterioration of the antioxidant defense mechanism leads to increased oxidative stress. Our results from the A549 cell line show that TOS levels were not significantly influenced by the viral infection compared to the control group. For the A549 cell line, the OSI was the most sensible parameter, and it was significantly higher than other studied parameters compared to the control A549 cells. Its higher value in infected cells indicates substantial oxidative imbalance [20]. Total antioxidant capacity (TAC) levels were reduced in all infected A549 cells, but the most significant reduction was observed for HCoV-229E and HRV A. For the human lung fibroblasts (MRC-5 cell line), the highest TOS values were noted for HCoV-229E, HAdV5, and HRV A when compared to the control cells.

The opposite effects were observed for the human lung carcinoma A549 cell line. Total antioxidant capacity was significantly higher in HCoV-229E and HRV A infected cells when compared to the control A549 cells. It is known that patients with SARS-CoV-2 infection who presented decreased TAC serum levels were more prone to disease exacerbation. In the case of those patients, the differences in TAC levels were attributed to high ROS production, an acute inflammatory condition, the infiltration of inflammatory cells into the different organs, multiorgan involvement, and declined oxygen saturation [21].

The oxidative stress index (OSI) is a ratio between TOS and TAS expressed in arbitrary units [22]. In the presented study OSI values were significantly increased in A549 cells after viral infections in all studied groups when compared to the control but not in MCR-5 cell lines. The studies evaluating systemic oxidative balance using the OSI in viral respiratory infections have been scarce. Increased systemic oxidative stress is related to disease severity in patients with respiratory diseases; thus, OSI levels can be a new predictor marker for the disease severity and used in managing the studied diseases.

Respiratory viruses not only increase oxidative stress marker levels and enhance ROS generation but also impair cellular defense mechanisms against them. While healthy levels of ROS play a crucial function in signaling, their persistently high levels result in significant oxidative damage. The antioxidant defense system comprises various enzymes and a range of low molecular weight compounds commonly known as antioxidants. These chemicals directly neutralize ROS, facilitate the recycling of defense enzymes, or modulate redox-sensitive transcription factors [23]. The targeted antioxidant markers include enzymes such as superoxide dismutases, catalase, peroxiredoxins, and glutathione peroxidases. Three isoforms of superoxide dismutase (SOD) transform oxygen into H_2_O_2_. Soluble peroxides are neutralized by catalase, peroxiredoxins, and glutathione peroxidases. Peroxiredoxins vary in their location and reactivation mechanisms. Also, GPx enzyme variants exhibit distinct localization patterns, in-cell activity, and different affinity for various types of peroxides. Glutathione peroxidases are selenium-dependent and -independent antioxidant enzymes that catalyze H_2_O_2_ reduction into water and oxygen [24]. The cytosolic selenium-dependent GPx is ubiquitously expressed throughout the body [24]. In the lungs, GPx is present in the epithelium, alveolar epithelial lining fluid, and alveolar macrophages [25]. A protective role of GPx has been demonstrated in various disease states involving ROS, including ischemia/reperfusion injury [26,27] and smoking [28].

Glutathione, a crucial antioxidant that neutralizes reactive oxygen species generated in host cells during RNA virus infections, modulates innate immunity at varying levels of viral infection [23,29]. Glutathione-dependent antiviral pathways appear to be pivotal in the immune response against viruses like influenza or rhinovirus [30]. The administration of glutathione in drinking water has been demonstrated to diminish viral titers in the mouse lung during influenza [31].

In the presented study, the A549 cell line presents less significant changes in enzymatic oxidative stress markers than in non-enzymatic oxidative stress markers. Glutathione peroxidase activity was significantly increased in the A549 cells infected by HAdV5, while in the MRC-5 cells, GPx activity was increased in cells infected with HCoV-229e and HCoV-OC43. In MRC-5 cells, infection with HRV A caused a significant depletion in the GPx activity when compared to the control cells. Infection with HAdV5 caused a significant reduction in GR activity in MCR-5 cells, while no significant differences were observed in the A549 cells when compared to control cells.

## 4. Materials and Methods

### 4.1. Study Objects

#### 4.1.1. Viruses

The study used the following viruses obtained from ATCC collection (Manassas, VA, USA): human coronavirus OC43 (VR-1558), human coronavirus 229E (VR-740), human adenovirus 5 (VR-1516), and human rhinovirus A (VR-1559).

#### 4.1.2. Cell Lines

The study used cell lines obtained from the ATCC collection (Manassas, VA, USA). Cell lines of lung carcinoma A549 (CRM-CCL-185) and lung fibroblast MRC-5 (CCL-171) were used for the experimental part of the study. Based on the available literature, it was determined that each of the tested viruses replicated efficiently in the chosen cell lines [32,33,34,35,36,37,38,39,40]. The study also involved using additional cell lines for virus stock preparation. The experiment was repeated 7 times for each virus and cell line used, which makes the number of samples significant for statistical analysis.

### 4.2. Test Virus Suspension Preparation

Each virus was cultivated on its default cell line. Coronaviruses OC43 and 229E were propagated on HT-29 cells (HTB-38), HAdV5 was cultivated on A549 cells, and HRV A on the HeLa line (CRM-CCL-2).

The cell lines used for test virus suspension preparation were cultivated in 175 mL flasks (NEST SCIENTIFIC Biotechnology, Woodbridge, NJ, USA). The Minimum Essential Medium with Earle’s BSS (Sartorius, Göttingen, Germany) for A549 and HeLa cell lines and Dulbeco’s Minimum Essential Medium with Earle’s BSS with an addition of 10% fetal bovine serum (FBS) (Sartorius, Göttingen, Germany) for HT-29 cell line were used as growing medium. The growing medium was removed, and cells were rinsed with PBS with the addition of magnesium and calcium ions (Sartorius, Göttingen, Germany) before the virus was added. Viruses’ stocks of 1 × 10^5^ concentration were added to the cell monolayer and incubated for 1 h at 37 °C with gentle shaking every 15 min. After 1 h, the virus stock suspension was removed, cells were rinsed again with PBS, and the fresh growing medium was added.

After the cells showed cytopathic effect (CPE), they were frozen at −80 °C and defrosted three times, followed by low-speed centrifugation (10 min, 1500× *g*) in order to sediment cell debris. The aliquots of test virus suspension were stored at −80 °C.

### 4.3. Test Cells Preparation

The experimental A549 and MRC-5 cells were inoculated on 6-well plates. They were propagated with Minimum Essential Medium with Earle’s BSS with the addition of 10% fetal bovine serum. When the cells reached a confluency of 80%, they were ready to be inoculated with viral test suspension.

### 4.4. Test Cells Inoculation with Viruses

The A549 and MRC-5 cells showing 80% confluency were infected with the test virus suspensions. Growing medium was removed from the wells, and the cells were rinsed with PBS. Then, 2 mL of the virus suspended in infection medium (Minimum Essential Medium with Earle’s BSS with addition of 1% fetal bovine serum) was added on the cells in each tested well and incubated with cells for 1 h with gentle shaking every 15 min. After incubation, virus suspension was removed, cells were rinsed with PBS, and fresh growing medium was added (Minimum Essential Medium with Earle’s BSS with addition of 10% fetal bovine serum). Infected cells were incubated up to 4 days until the CPE could be seen.

### 4.5. Preparing Cells for Oxidative Stress Marker Measurements

The infected and the control cells were collected for further examinations when the viral CPE became visible. Growing medium was removed from each well and collected in 15 mL sterile centrifuge tubes. Cells attached to the surface of wells were treated with trypsin (Gibco™, Thermofisher Scientific, Waltham, MA, USA) and collected in 15 mL sterile centrifuge tubes. All samples were centrifuged at 1800 rpm for 10 min (Frontier™ Series 5000 Multi-Pro, OHAUS Europe GmbH, Nänikon, Switzerland). Supernatants were removed, and the obtained cells were suspended in PBS (without calcium and magnesium ions) and stored at −80 °C for further examination.

### 4.6. Oxidative Stress Markers

All oxidative stress markers were assayed using spectrophotometrical methods. The changes in absorbance were measured on a PERKIN ELMER Victor X3 reader (Perkin Elmer, Inc., Waltham, MA, USA).

#### 4.6.1. Total Oxidative Status (TOS)

Total oxidative status (TOS) was determined using Erel’s method [13]. The method measures the color intensity of Fe3+ ions and xylenol orange complex in an acidic environment. TOS was expressed as µM of H_2_O_2_ (standard) per mg protein of the tested sample.

#### 4.6.2. Total Antioxidant Capacity (TAC)

Total antioxidant capacity (TAC) was measured using method by Erel [41]. In this colorimetric method, a colorless reduced 2,2′-azino-bis(3-ethylbenzothiazoline-6-sulfonate) (ABTS+) is oxidized to blue-green ABTS+, which when mixed with any substance that can be oxidized, is reduced to its original colorless reduced form. TAC was expressed as µM of Trolox (standard) per mg of protein of the tested sample.

#### 4.6.3. Oxidative Stress Index (OSI)

The oxidative stress index (OSI) was calculated as the percentage of TOS-to-TAC ratio [20].

#### 4.6.4. Glutathione Peroxidase (GPx) Activity (EC 1.11.1.9)

Glutathione peroxidase (GPx) activity was measured using the kinetic method by Oyanagui [42]. During the reaction, the oxidized glutathione (GSSG) is formed from GSH produced by glutathione reductase (GR) present in the reaction mixture. The GPx activity in the assessed samples was expressed as μmoles of nicotinamide adenine dinucleotide phosphate (NADPH) oxidized in 1 min per 1 mg of protein (IU/min/mg protein).

#### 4.6.5. Glutathione Reductase (GR) Activity (EC 1.8.1.7)

Glutathione reductase activity was determined using the kinetic method by Aebi [43]. The method is based on changes in the concentration of NADPH that reacts with oxidized glutathione (GSSG) to form GSH. The activity of GR in the assessed samples was expressed as μmoles of NADPH utilized in 1 min per 1 mg of protein (IU/min/mg protein).

### 4.7. Statistical Analysis

The normality of the distributions was assessed using the Shapiro–Wilk test. Data with normal distributions were presented as mean ± standard deviation (M ± SD). Data with skewed distribution were presented as median with lower quartile and upper quartile (Me(Q_1_; Q_3_)). The homogeneity of variance was assessed using Levene’s test. Comparisons of oxidative stress markers in virus-infected cells were made using ANOVA followed by Dunnett’s test or, if assumptions were not met, the Kruskal–Wallis ANOVA test. All tests used were two-tailed. The results were considered statistically significant at *p* < 0.05. Calculations were performed using Statistica (data analysis software system version 13 (TIBCO Software Inc. 2017, Palo Alto, CA, USA)).

## 5. Conclusions

We observed that respiratory viral infection more significantly affected non-enzymatic oxidative stress markers in a lung adenocarcinoma model (A549 cells), while human lung fibroblasts (MRC-5 cell line) presented changes in enzymatic and non-enzymatic oxidative stress markers. We suggest that further detailed research is required to analyze this phenomenon.

## Data Availability

Data available after contact with corresponding author.

## Supplementary Materials

The following supporting information can be downloaded at https://www.mdpi.com/article/10.3390/ijms252212088/s1.
