# Supplementary material for: The Effects of Different Respiratory Viruses on the Oxidative Stress Marker Levels in an In Vitro Model: A Pilot Study
Source: Int J Mol Sci. 2024 Nov 11;25(22):12088. doi: 10.3390/ijms252212088 (PMC11593713; doi:10.3390/ijms252212088)
Supplement: Supplementary file 1 [file ijms-25-12088-s001.zip › ijms-3255459-supplementary.pdf]

Effects of different respiratory viruses on the oxidative stress markers levels in in vitro model: A pilot study

Barbara Bażanów [1], Katarzyna Michalczyk [2], Alina Kafel [3], Elżbieta Chełmecka [4], Bronisława Skrzep-Poloczek [2], Aleksandra Chwirot [1], Kamil Nikiel [2], Aleksander Olejnik [2], Alicja Suchocka [2], Michał Kukla [5,6], Bartosz Bogielski [2], Jerzy Jochem [2] and Dominika Stygar [2,*]

Table S1. Mean values of oxidative stress markers in the cells infected with different respiratory viruses and in control cells of the human lung carcinoma cell line (A549). Results are presented as Me (Q_1;Q_3) – median (lower-upper quartile) or M ± SD – mean ± standard deviation.

Variables	Control A549	HCo-OC43	HCo-229E	HAdV5	HRV A	p_{ANOVA}
TOS (μM H_2O_2/mg protein)	22.2 (12.6;34.5)	21.7 (20.6;26.7)	14.6 (14.0;15.9)	14.8 (14.5;14.9)	13.5 (13.1;13.8)	< 0.01
TAC (μM Trolox/mg protein)	77.8 (75.1;79.9)	6.4 (54;7.1)	5.1 (4.8;5.3)	7.3 (6.7;7.9)	5.2 (5.1;5.5)	< 0.001
OSI (%)	29.3 ± 13.0	352.5 ± 35.7	303.0 ± 36.6	201.4 ± 14.0	254.1 ± 14.9	< 0.001
GPX (IU/min/mg protein)	0.34 (0.33;0.47)	0.58 (0.38;0.60)	0.40 (0.39;0.41)	0.74 (0.72;0.79)	0.29 (0.21;0.35)	< 0.001
GR (IU/min/mg protein)	0.721 (0.689;0.860)	0.990 (0.937;1.099)	0.068 (0.067;0.073)	0.054 (0.052;0.053)	0.053 (0.051;0.053)	< 0.001

Legend: GPx – glutathione peroxidase activity, GR – glutathione reductase activity, HAdV5 – human adenovirus 5; HCoV-229E – human coronavirus 229E, HCoV-OC43 – human coronavirus OC43, HRV A- human rhinovirus A, IU – international activity unit; OSI – oxidative stress index, TAC – total antioxidants capacity, TOS – total oxidative status.

Table S2. Mean values of oxidative stress markers in the cells infected with different respiratory viruses in the lung fibroblasts cells line (MRC-5). Results are presented as Me (Q_1;Q_3) – median (lower-upper quartile) or M ± SD – mean ± standard deviation.

Variables	Control MRC-5	HCoV-OC43	HCo-229E	HAdV5	HRV A	p_{ANOVA}
TOS (µM H2O2/mg protein)	10.9 ± 0.5	5.2 ± 0.2	66.1 ± 2.1	31.4 ± 0.5	66.2 ± 1.0	< 0.001
TAC (µM Trolox/mg protein)	22.4 (19.2;28.4)	35.4 (34.8;35.9)	25.8 (22.8;30.0)	36.1 (32.9;38.1)	80.3 (80.0;80.6)	< 0.001
OSI (%)	46.1 (44.6;48.7)	14.5 (12.5;17.1)	246.1 (223.3;278.2)	87.4 (76.4;168.1)	82.7 (81.8;83.0)	< 0.001
GPX (IU/min/mg protein)	0.79 ± 0.04	1.70 ± 0.19	2.35 ± 0.30	1.13 ± 0.08	0.19 ± 0.00	< 0.001
GR (IU/min/mg protein)	0.028 (0.025;0.036)	0.028 (0.026;0.029)	0.022 (0.021;0.023)	0.016 (0.015;0.016)	0.019 (0.018;0.019)	< 0.001

Legend: GPx – glutathione peroxidase activity, GR – glutathione reductase activity, HAdV5 – human adenovirus 5; HCoV-229E – human coronavirus 229E, HCoV-OC43 – human coronavirus OC43, HRV A- human rhinovirus A, IU – international activity unit; OSI – oxidative stress index, TAC – total antioxidants capacity, TOS – total oxidative status.
